# Clinical Holistic Medicine: How to Recover Memory Without “Implanting” Memories in Your Patient

**DOI:** 10.1100/tsw.2007.237

**Published:** 2007-09-17

**Authors:** Søren Ventegodt, Isack Kandel, Joav Merrick

**Affiliations:** ^1^ Quality of Life Research Center, Teglgårdstræde 4-8, DK-1452 Copenhagen K, Denmark; ^2^ Research Clinic for Holistic Medicine, Copenhagen, Denmark; ^3^ Nordic School of Holistic Medicine, Copenhagen, Denmark; ^4^ Scandinavian Foundation for Holistic Medicine, Sandvika, Norway; ^5^ Interuniversity College, Graz, Austria; ^6^ Faculty of Social Sciences, Department of Behavioral Sciences, Ariel University Center, Samaria, Ariel, Israel; ^7^ National Institute of Child Health and Human Development, Jerusalem, Israel; ^8^ Office of the Medical Director, Division for Mental Retardation, Ministry of Social Affairs, Jerusalem, Israel; ^9^ Kentucky Children's Hospital, University of Kentucky, Lexington, USA

**Keywords:** holistic medicine and health, clinical holistic medicine, CAM, psychodynamic therapy, symbolic incest, energetic incest, implanted memories, implanted philosophy, holistic sexology, bodywork, vaginal acupressure

## Abstract

Every therapeutic strategy and system teach us the philosophy of the treatment system to the patient, but often this teaching is subliminal and the philosophical impact must be seen as “implanted philosophy”, which gives distorted interpretations of past events called “implanted memories”. Based on the understanding of the connection between “implanted memory” and “implanted philosophy” we have developed a strategy for avoiding implanting memories arising from one of the seven most common causes of implanted memories in psychodynamic therapy: 1) Satisfying own expectancies, 2) pleasing the therapist, 3) transferences and counter transferences, 4) as source of mental and emotional order, 5) as emotional defence, 6) as symbol and 7) from implanted philosophy. Freud taught us that child sexuality is “polymorphously perverted”, meaning that all kinds of sexuality is present at least potentially with the little child; and in dreams consciousness often go back to the earlier stages of development, potentially causing all kinds of sexual dreams and fantasies, which can come up in therapy and look like real memories. The therapist working with psychodynamic psychotherapy, clinical holistic medicine, psychiatry, and emotionally oriented bodywork, should be aware of the danger of implanting philosophy and memories. Implanted memories and implanted philosophy must be carefully handled and de-learned before ending the therapy. In conclusion “clinical holistic medicine” has developed a strategy for avoiding implanting memories.

## INTRODUCTION

During the last decade there has been an intense and ongoing debate in the medical scientific community about therapy and implanted memories[1,2]. It has generally been concluded that memory is not perfect and often more like an idea or an impression than actually like a movie that you can play again and see what really happened. Memory in this sense is known to be highly sensitive to emotions and expectations, as is well known from forensic psychology. Another problem is that the human being constantly has fantasies and reveries[3,4], and when we remember such a fantasy, this is an actual remembrance but of an unreal event. If this happens with a patient, this can cause large confusion in therapy. In general the mind is not very reliable and the interpretation of the world in present time and in the past seems to be easily affected by intentions and needs, both bodily and mental.

Because of this vagueness of most people's memories it is now generally believed that it is actually possible to implant "memories" during therapy. The normal solution in therapy is to be sure that you do not make any judgements about what actually happened, until the patient finds out for herself what happened. It is important to actively avoid influencing the process of interpretation (i.e. give suggestions that can be taken as indications of how a feeling or gestalt should be interpreted by the patient). The central dogma of not interpreting the material of the patient is at the root of classical psychoanalysis, and gives a relaxed and often not-so-intense kind of therapy that often includes several hundreds of hours of therapy during several years.

When it comes to intensive psychodynamic short-term psychotherapy (often defined as less that 40 sessions) and existential psychotherapy the therapist becomes more dependent of his own theory for the individual patient[5,6]. Unfortunately, the patient will often know this theory, or sense it as the therapist cannot help revealing its central idea in the way he approaches the patient, and the subjects he addresses in the therapy. In the beginning of the therapy the only way the patient can cooperate is letting go of the control and playing along. In doing this there is a lot of learning that is actually *implanting philosophy*. When the patient's personal past is seen in the light of this new or corrected philosophy, the whole past will look different, which is actually also the core idea of therapy. So every therapist is in fact *implanting memories* in the broadest sense of this concept.

When the therapist expects sexual abuse to be the caboose of a complex of symptoms, the patient will look for and often find events that can be interpreted in this way, in order to comply. Here we have the *implanted memories* of incest or abuse. The problematic thing about such memories is that if they are taken as real, the patient needs to "clear" the relationships with the relevant people (often the parents or other family members), and often this is done in a non-forgiving and destructive way harming the patient and sometimes also her surroundings. The loss of self-esteem in connection with such a recovery of incest memories is always a difficult problem, but can be solved in existential therapy. If the events are implanted memories, incongruence is introduced, making it very difficult for the patient to move forward, and heal herself and her relationships to the people of her world. (Please notice that we use "she" as the sexually abused patient is normally a woman, but the patient could as well be a man; we use "he" about the therapist who is sometimes a man but could as well be a woman).

This becomes even more problematic, when intensive psychodynamic short term psychotherapy are combined with bodywork and holistic gynaecological/sexological therapy[7-18], where the intensity of the confronted repressed emotions in the therapy often is getting high. The reason for using the combination of techniques is that the patient needs a lot of support on many different levels, to be able to confront i.e. a childhood rape scenario without experiencing unbearable existential pain in the session.

We have worked with the problem of how to avoid implanted memories for years in the research project "Quality of life and aetiology of diseases" and believe that we have come to a practical solution of the problem, allowing us to make the most intensive therapy without damaging the patients (i.e. by implanting memories). A recent follow-up of 109 patients from our Research Clinic for Holistic Medicine in Copenhagen after clinical holistic medical treatment (receiving the mindful combination of psychodynamic short time therapy and bodywork) has documented that the patients were not harmed, but often helped by this therapy[19-24]. A pilot study of 20 women that had continuous sexual problems on average for almost nine years (in spite of seeing physicians and alternative therapists over that period) showed that most of the patients were helped in this therapy and no patient was harmed (reporting significant side-effects or ending at a lower score in quality of life, health and ability that before starting the therapy)[17].

We have solved the problem on a theoretical level, and when we took this solution into practice we found that it worked good with reliable results. We used contemporary models from the research fields of quality of life, human development, and holistic medicine to understand what happened in therapy to make implanted memories possible. We found a simple solution to the complex problem of implanted memories, which is recovering the memory and sense of truth in general in the patient.

## Seven Causes of Implanted Memories

The seven most common causes of implanted memories are:

*1)* *Satisfying own expectancies:* If the patient expects that she had been abused sexually i.e. because a sister was, she can implant more or less vague memories of incest herself.

2) *Pleasing the therapist*: The patient wants to be in accordance with the therapist and is therefore accepting his view or what she believes or imagine is his view. This is enhanced if the therapist shares his interpretations and gives the patient leads (i.e. questions that are not neutral but biased in some direction); and even more if the therapist is making judgments on what happen in stead of bearing not knowing what happened until the patient finds our for herself.

3) *Transferences and counter transferences:* If the patient develops sexual feelings towards the therapist and if these are ignored by the therapist, or if the patient senses that the therapist will not accept them, this can enhance sexual fantasies, which eventually can take the form as an implanted memory; old sexual fantasies can also be boosted by this unconscious wish in the patient, and even real events can be distorted and reinterpreted now filled with the sexual feelings that the patient cannot allow to emerge in the personal relationship to the therapist.

4) *As source of mental and emotional order:* A third source of implanted memories has nothing to do with the therapy in itself. The patient needs to get a kind of order in the chaos of emotions and symptoms, and having a simple explanation can be a relief instead of living with chaos and mystery.

*5)* *As emotional defence.* Sometimes the recovered but false memory is hiding another event that is much more painful. This could be that her father left her and her mother when she was a child. This may be much more difficult to integrate than sexual abuse. If the patient is desperately angry with her father and cannot confront the event causing the anger, an implanted event can be a solution. It could also be neglect that is the problem; it seems that neglecting the bodily presence and sexual character of a girl can be as destructive to her self-esteem and psychosexual development as actual physical or sexual abuse.

*6)* *As symbol.* Often, the parents have been abusing the child in subtle and psychological ways, (i.e. not respecting the child’s sexual borders, or having used the child as a sexual partner, which is most often seen when a parent lives alone with a child of the opposite sex). This does not mean that there was a sexual act of objective, physical, incest like coitus, but what we could call the “symbolic incest” or “energetic incest” is often extremely painful and very harmful to a child on an emotional level. “Energetic” incest happens typically when her father being the only parent raises a girl (or when a mother raises her son alone), and the two of them “pair up” as man and woman making wholeness emotionally and energetically comparable to the wholeness of a sexual couple, but without the sexual acting out. A lot of sexual energies are accumulated and circulated here, and the girl is often, as Freud pointed out, having secret sexual dreams about her father with lots of shame and guilt. An implanted memory that carries all the shame and energy of a real incestuous trauma, but where intense therapy do not reveal any recorded "movie" of the event(s), might very well come from "energetic incest".

7) *Implanted philosophy*. When a patient learns that problems often are caused by traumas, she often starts speculating which traumas could have caused which problems. Sexual problems often then lead to dreams about sexual dominance/abuse/perversions and dreams can be interpreted as memories. Freud taught us that the child's sexuality is "polymorphously" perverted, meaning that all kinds of sexuality is present at least potentially with the little child. In dreams, according to Freud, consciousness often goes back to the earlier stages of development, potentially leading to all kinds of sexual dreams and fantasies.

The mind can interpret the same event in many different ways and one version of a "memory" cannot immediately be trusted over others. Many therapists therefore turn to the physical body for the truth about the past of the patient, assuming that the body cannot lie, because it carries the traumas as tensions that can be released, when the emotional and cognitive content of the gestalt is reintegrated in the consciousness of the patient. But as the body is seen through the patient's delusive mind, just turning from the mind to the body does not solve the problem of validating that a particular event actually happened as the patient recalls it.

## The Three Phases of Existential Holistic Therapy and The Arch of Metamorphosis

During the last decade of research in clinical holistic medicine at the Research Clinic for Holistic Medicine in Copenhagen, we have found that the therapy in general has three phases[25-28]:

1) Feeling the repressed emotions of the past
2) Understanding the objective elements of the traumatic event
3) Modifying/changing negative beliefs about the traumatic event ("letting go")

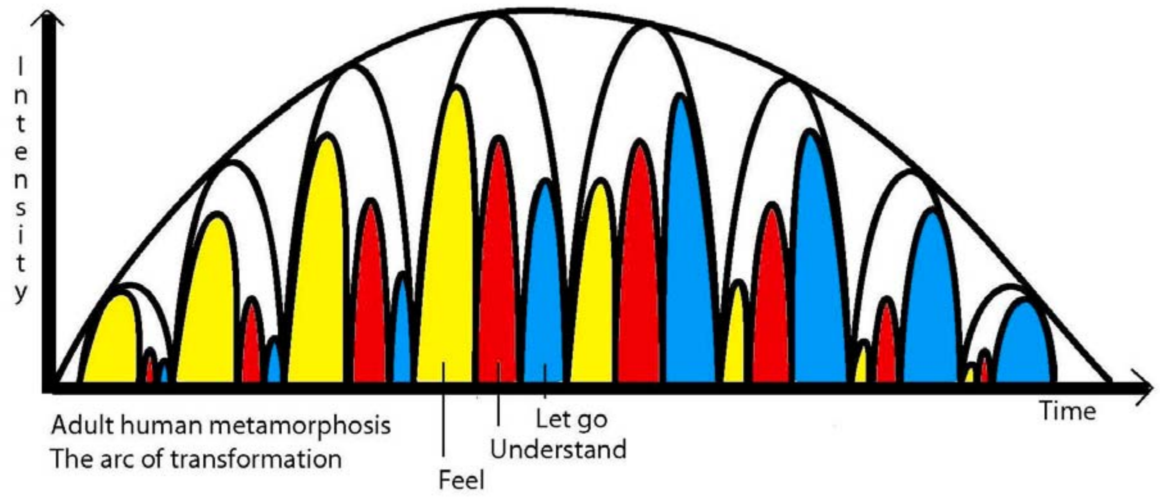

**Figure 1:** The arch of therapeutically transformation in clinical holistic medicine. There is three core elements of the therapeutic process: to feel (yellow), to understand (red), and to let go (blue) of negative, life-denying beliefs and attitudes. In the first sessions the emotional discharge dominates; as intensity in therapy grows, the element of understanding becomes more dominant, and in the end when the "heat" leaves therapy, cool understanding raises from the bottom of the patients soul (wholeness)[15].

We have analysed the therapeutic work of about 500 patients with a number of different diseases and health issues[29-45] and learned that, in general, therapy has the following course. In the first sessions, the emotional discharge dominates; as intensity in therapy grows, the element of understanding the traumatic event becomes dominant, and in the end when the intensity leaves the therapeutic process, a deeper understanding arises from the bottom of the patient's soul (wholeness).

We have also learned that the therapeutic process can be understood as a metamorphosis – the patient enters the therapy like a butterfly's larvae in need of transformation; she lets go of her old identity and melts down (entering the "pupae"). In this state, she develops a new understanding from recalling what she was originally meant to be; and finally she enters the world again as a renewed and transformed person (free to fly like the butterfly), much more beautiful, good and true. We know this process as the autogenetic process (see Antonovsky [46,47]), where the patient regains physical and mental health, quality of life, and the ability to function in all areas of life. During this process, the patient will experience a number of crises that are not dangerous to the patient assuming that the patient is cared for intensively and properly (see figure 2).

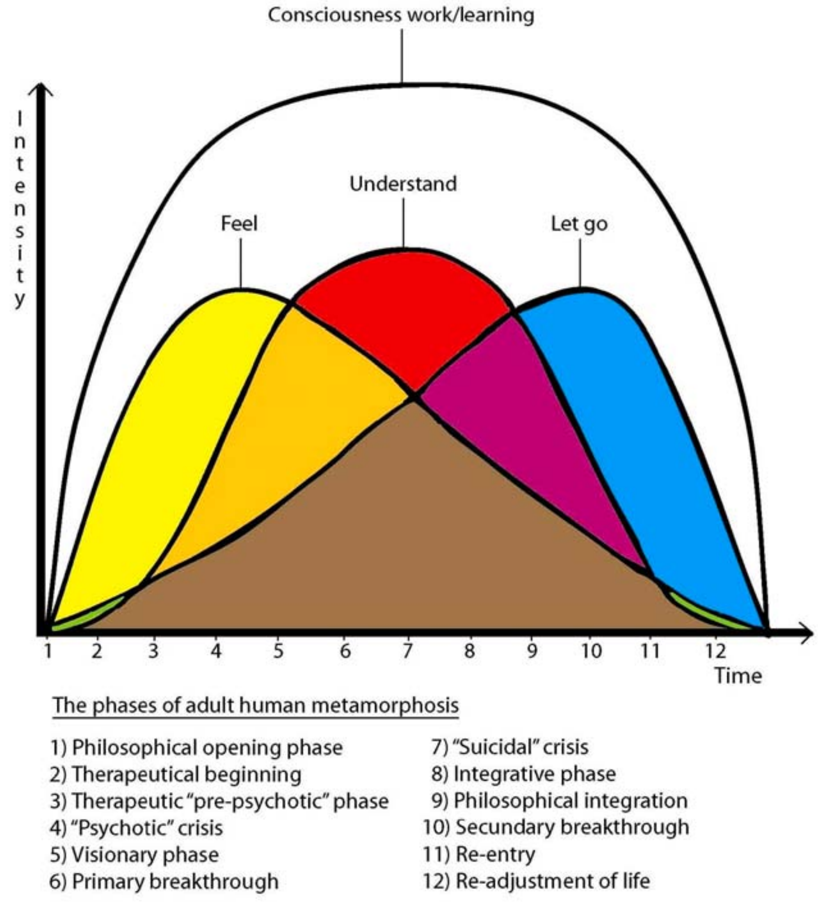

**Figure 2:** The experimentally found major phases and crisis in intensive dynamic short time therapy when complemented with bodywork [from 15].

## A Model for The Wholeness of Man

Humans have classically been described as consisting of three separate entities, all of which in psychoanalytical therapy are seen as carrying each a very different representation of self: the body carrying the Id, the mind carrying the Ego, and the wholeness carrying the True Self (higher self, soul, comparable to Freud's "Super Ego"). The wholeness of man consist of these three parts, and this points to a simple reason why neither the body nor the mind can be trusted much: they are only parts of our being, and as such they are not able to contain the totality. Only through our wholeness can we truly "see" the world and our personal history.

This understanding is very important as it gives us a key to understanding why patients cannot remember much in the beginning of the therapy, when they are starting to confront their own emotions. We also understand the reason for the intensive involvement of the mind in the second phase of the therapy, which does not provide clear understanding and recalling (memory) to the patient. It is only in the third phase of therapy, when the patient lets go of all negative and defensive beliefs and attitudes and returning to her natural philosophy of life will everything become clear, and (s)he will find herself remembering and understanding everything as the "true" reality..

Most interestingly, the majority of patients may see themselves as part and parcel of a severely harmed body and not a free and enjoyable spirit (the wholeness, the free and true "soul"). In therapy, the patient needs first to recover the energy of the body (physical character and sexuality - a process, which has been used as medicine since Hippocrates) then they need to recover the mind (the mental character), and finally they will recover the spiritual dimensions of love, individual talents, higher intelligence (the spiritual character and purpose of life/life mission), and real happiness coming from being able to contribute to the world.

In the therapy we often teach this in a popular way to the patients, talking about "the four doors of existential therapy":

1) getting into sexuality
2) getting into consciousness
3) getting into love
4) getting into life

Most (Danish) patients realize the needs of re-conquering these dimensions of life, and therefore understand and accept this path to the healing of existence (salutogenesis)[46,47,48] immediately. Interestingly, as this process proceeds, first ability to feel, then ability to understand, and finally ability to judge what is true and what is not comes into focus. This originates from the patients reconnecting to the universe, and obtaining the Antonovsky's existential experience of the *sense of coherence*[46,47,49].

## CASE EXAMPLE

A 24 year-old psychology student, very intelligent, with a "head-centred", mental approach to the world and with a strongly repressed sexuality presents in clinical holistic therapy desiring to solve her existential and sexual problems. She strongly expects her father to have abused her sexually and remembers many such events. As therapy progresses and the emotional charge is relieved, she gradually changes her mind about the occurrence of abuse. After the 5 first sessions, she starts to doubt that she has been physically abused, and in the end she realises the sexual abuse to be energetic (symbolic). Before the therapy she rated herself as functioning poorly sexually, with lack of sexual interest and orgasm, but after the integration of the energetic incest she becomes able to get a boyfriend that she chooses and a satisfying sex life. She manages to keep this relationship vital for years.

Intensive psychodynamic short-term psychotherapy with role-playing (re-parenting) and bodywork (body dynamics, vaginal acupressure) was used with this patient using the advanced therapeutic toolbox (see [12]). In the beginning of the therapy emotions were not intense with this patient, but only slowly did she open up. When she finally did, the session was almost exploding in intensity. The breakthrough session happened at point 6 in figure 2, right when the most intense feelings were turned into understanding. At this point in time, the realistic memories of the abuse are still hidden from the soul, and the mind can interpret such events in many different ways. The repressed sexuality of this patient seemed to distort the patient's memory up to and including sexual sadism. Most interesting was the therapeutic catharsis and the effect of allowing the patient to go fully into exploring her past history of sexual abuse, making her finally doubt that it really happened: "I can't understand that this should really have happened". In the session the patient was sent back into the early events using the principle of similarity. The issue related to using similarity is that you cannot, as a therapist, avoid "implanting the memory" that the patient and you as a physician agree upon treating. But in this phase the trauma cannot be remembered, because the emotional charge is efficiently blocking the admittance to the time line. So we are really making a drama, only led by the emotional charge of the patient's repressed traumas. But only by supporting the patient in confronting these emotions can she get closer to a real memory of what has happened to her. This is a most difficult technique that only can be done, when there is a very close and intimate relationship between the patient and the physician. At the same time, this intimacy invites implanted memories of the "transferences and counter transferences" kind (see above point 3 in causes of implanted memory). The situation looks impossible but fortunately the processing of the trauma and the subsequent emotional discharge is, in the end, the key to solving the problem. The only thing the therapist cannot do is to back out and abandon the patient.

A most interesting thing to observe in this example is the high degree of certainty she has about past traumatic events at the beginning of the session. When the memories start to clear up, after she has confronted the unbearable emotions of the gestalts, she becomes more and more doubtful that what she has remembered is "real". After reflecting deeply over the content of the session for some months she concluded that the abuse had not actually happened on physical level although it did happen energetically. Thus it was a symbolic representation of energetic abuse (the 6$^{th}$ reason of implanted memories, see above).

## DISCUSSION

The uses of the similarity principle with patients that believe that they have been sexually abused sometimes reveal that what they seem to remember and recover in the therapy did not actually happen. This is an amazing process of recovering severe sexually traumatic memories and through careful evaluation, the patient realizes that something completely different and much more complex actually happened.

We are complicated beings with needs and consciousness of many layers. As we develop, we need to be physically touched and emotionally supported, met at our borders and loved unconditionally. Unfortunately, most parents are not really able to meet the demands of their children and almost all children ends up more or less traumatized – a sad fact known ever since Freud.

The only way to cure somatic, mental, existential, and sexual problems arising from early childhood trauma is to discharge the emotional components by confronting the content of the traumas. The emotional charge also makes the trauma impossible to remember; the only route for inducing healing of the patient's existence (salutogenesis)[46,47,48] is to support a "blind" confrontation of the repressed emotional content of the patient's sub-consciousness. The similarity principle seems most useful[49-55], as this principle allows the therapist to take the patient directly down to confronting the old traumas causing the problems.

The problem with this kind of therapy has been the fear of planting memories by the therapy itself. Our experience with holistic existential therapy is that sometimes such false memories are in fact implanted, but as therapy progresses these implanted memories are seen as not true. This is happening when the patient acquires a soul-perspective and becomes able to look at the whole life – the whole timeline from conception to now – as one single event, that is understandable in the light of the purpose of life that then is denied and repressed (see the life mission theory[56-62]).

The only real problem with this form of therapy is if the patient drops out of the therapy before the temporarily false memories are reinterpreted and integrated. It is the obligation of the therapist to continue the therapy, until the patient is cured and free of her problem. Therefore, it is important that the patient stays in therapy no matter how unpleasant emotionally it is to confront the old traumas.

## CONCLUSIONS

We have analysed the problem of implanted memories and found that such implantations indeed do happen in therapy for a number of reasons. When extreme memories of sexual abuse occur in patients with a strongly repressed sexuality and a very active mind, the therapist should consider if the memories are actually implanted. This does not mean that he should disrupt the therapy, but he should most carefully be sure not to interpret for the patient. This allows the patient to modify the memories about what has really happened in her childhood. If the emotional charge of the early traumas – often feelings of guilt and shame – is systematically relieved, the patient will in the end obtain the position of being able to review her whole time line and understand the real events (no matter how traumatic) leading to the emotional charge that has given the patient so many challenges.

Only when the patient can look from her wholeness, the truth can be perceived and the past truthfully remembered. The therapist must be extremely certain that the therapy reaches this conclusion. Fictive memories temporarily implanted are not a problem if this happens, but will be if the therapy for some reason is disrupted.

Using the similarity principle [49-55] during intensive, mindful psychodynamic short term psychotherapy complemented with bodywork seems to be the most direct way to induce holistic healing – salutogenesis – in patients with a complex of somatic, mental, existential and sexual problems. The therapy will often be very intense and the content of the therapy might be extremely explicitly sexual. If the therapist can contain the patient and all her emotions, the existential healing can be completed with no serious hindrances.

Freud taught us that the child sexuality is “polymorphously” perverted, meaning that all kinds of sexuality is present at least potentially with the little child; and in dreams consciousness often go back to the earlier stages of development, potentially causing all kinds of sexual dreams and fantasies, which can come up in therapy and look like real memories. The therapist working with psychodynamic psychotherapy, clinical holistic medicine, psychiatry, and emotionally oriented bodywork, should be aware of the danger of implanting philosophy and memory. Implanted memories and implanted philosophy must be carefully handled and de-learned before ending the therapy. In conclusion, “clinical holistic medicine” has developed a strategy for avoiding implanting memories.

## ACKNOWLEDGEMENTS

This study was supported by grants from IMK Almene Fond. The quality of life research was originally approved by the Copenhagen Scientific Ethical Committee under number (KF)V.100.2123/91 and later correspondence.
